# Evaluation of the palatal bone in different facial patterns for orthodontic mini-implants insertion: A cone-beam computed tomography study

**DOI:** 10.1590/2177-6709.26.1.e2119204.oar

**Published:** 2021-03-22

**Authors:** José Antonio VIDALÓN, Carlos LIÑAN, Lidia Yileng TAY, Abraham MENESES, Manuel LAGRAVÈRE

**Affiliations:** 1 Peruvian Cayetano Heredia University, Faculty of Dentistry, Department of Orthodontics (Lima, Peru).; 2 University of Alberta, Graduate Program of Medicine and Dentistry (Edmonton, Canada).; 3 Peruvian Cayetano Heredia University, Faculty of Dentistry, Master Program in Orthodontics (Lima, Peru).

**Keywords:** Cone-beam computed tomography, Orthodontic anchorage procedure, Palate

## Abstract

**Objective:**

Evaluate the height, thickness and cortical density of the palatal bone of adults with different vertical facial patterns using Cone-Beam Computed Tomography (CBCT).

**Methods:**

This study analyzed 75 CBCTs of patients between 18 and 35 years old (45 men and 30 women). The CBCTs were classified into three groups based on their facial pattern: normodivergent, hypodivergent and hyperdivergent as determined from lateral cephalograms synthesized from the CBCTs. The height, cortical thickness and cortical density of the palatal bone were measured at 4, 8, 12, 16 and 20mm posterior to the incisive foramen, and at 3, 6 and 9mm lateral to the midpalatal suture. ANOVA with Tukey *post-hoc* tests were used for analysis of the data, at significance level of *p*< 0.05.

**Results:**

The hypodivergent pattern had a significant difference and the greatest height and cortical thickness of the palatal bone, followed by the hyperdivergent and the normodivergent patterns. No significant differences were found in minimum and maximum values of cortical density.

**Conclusion:**

The palatal bone is a favorable anatomical area to install different orthodontic temporary anchorage devices (TADs), where individuals with the hypodivergent vertical facial pattern have a higher height and cortical thickness of the palatal bone, followed by the hyperdivergent pattern and finally the normodivergent pattern. No significant differences in the cortical density of the palatal bone in the three facial patterns were found.

## RESUMO

**Objetivo:**

Avaliar a altura, a espessura e a densidade cortical do osso palatino em adultos com diferentes padrões faciais verticais, utilizando a tomografia computadorizada de feixe cônico (TCFC).

**Métodos:**

O presente estudo analisou 75 TCFCs de pacientes com idades entre 18 e 35 anos (45 homens e 30 mulheres). As TCFCs foram classificadas em três grupos, de acordo com seus padrões faciais: normodivergentes, hipodivergentes e hiperdivergentes, conforme determinado na radiografia cefalométrica lateral reconstruída das TCFCs. Altura, espessura e densidade cortical do osso palatino foram aferidas a 4, 8, 12, 16 e 20 mm para posterior do forame incisivo e a 3, 6 e 9 mm lateralmente à sutura transpalatina. Os testes ANOVA e *post-hoc* de Tukey foram utilizados para análise dos dados, com nível de significância de *p*< 0,05.

**Resultados:**

O padrão hipodivergente apresentou uma diferença significativa e a maior altura e espessura cortical do osso palatino, seguido pelos padrões hiperdivergente e normodivergente. Nenhuma diferença estatisticamente significativa foi encontrada nos valores mínimos e máximos da densidade cortical.

**Conclusão:**

O osso palatino é uma área anatomicamente favorável para instalar diferentes dispositivos de ancoragem temporária. Indivíduos com padrão facial vertical hipodivergente apresentam maior altura e espessura cortical do osso palatino, seguido do padrão hiperdivergente e finalmente do padrão normodivergente. Não foi encontrada qualquer diferença significativa na densidade cortical do osso palatino entre os três padrões faciais.

## INTRODUCTION

During orthodontic treatment, teeth are exposed to forces and moments generated by the appliances used. The applied forces generate reciprocal forces of the same magnitude in the opposite direction. Thus, one of the most difficult clinical challenges is to minimize these reciprocal forces. Successful treatment generally depends on meticulous planning of the anchorage.^1^ A reliable method is to use temporary anchorage devices (TADs).

The palatal region is very important for the installation of TADs as an aid in the orthodontic treatment, showing a high clinical versatility, with more precise and predictable tooth movement regardless of patient cooperation.^2^ ^,^ ^3^

One factor determining the success of TADs placement is the quantity of the surrounding bone.^4^ The insertion on the palate depends on the structural characteristics of the palatine bone, such as height, cortical thickness and cortical density. It has been reported that a suitable bone thickness of the palate should be greater than 4 mm.^5^ Bone characteristics can be evaluated through the cone-beam computed tomography (CBCT), which provides highly accurate and detailed information.^6^ ^,^ ^7^

The skeletal morphology in the craniofacial region is primarily controlled by genetic factors. However, the functional demands can have a significant effect on the growth and craniofacial development. Each of the facial patterns in the vertical dimension (hyperdivergent, normodivergent and hypodivergent) present differences in the muscle load during function, due to skeletal compensation. This muscle load can alter the height and thickness of the cortical bone and the density of the palatal bone, not only in muscle attachment sites but also in other skeletal sections.^8^ It could be said that there is a significant relationship between the facial type and the morphological characteristics of the jaws.^9-11^ Sadek et al.^10^ reported that patients with a hyperdivergent pattern have a narrow alveolus, compared to the normal and hypodivergent patients.

The aim of this study was to determine the height, thickness and cortical density characteristics of the palatal bone in the different vertical facial patterns using CBCTs. This information would give background or guidelines in terms of possible TAD placement sites in the palatal bone dependent on the patient growth pattern.

## MATERIAL AND METHODS

**Figure 1:**
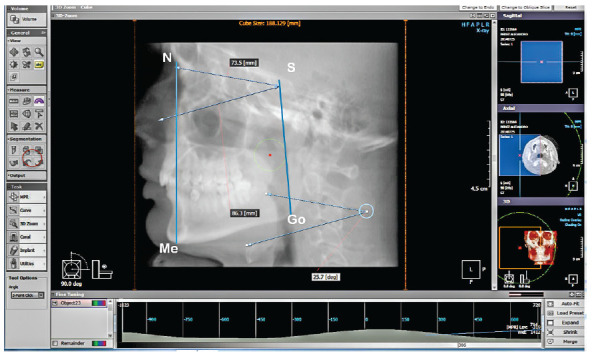
Measurements of facial patterns: 1) Anterior cranial base (sella [S] to nasion [N]) and mandibular plane (gonion to menton), 2) Face height index, the ratio of posterior face height to anterior face height using the measurements of distance from sella (S) to gonion (Go) divided by the distance of nasion (N) to menton (Me).

This study analyzed 75 CBCTs (25 normodivergent, 25 hypodivergent and 25 hyperdivergent), from patients between 18 and 35 years old, with permanent dentition and in maximum intercuspation (45 men and 30 women).The sample size formula was applied to estimate an average: n= 2(Z_α_+Z_β_)^2*^S^2^/d^2^. Patients with facial asymmetries, hyperplasia and obvious craniofacial syndromes, cleft lip and palate, systemic diseases, and presence of impacted teeth in the palatal region were excluded. Subjects were classified into one of three groups, based on their vertical facial pattern and with no sagittal malocclusion, as determined from lateral cephalograms synthesized from the CBCTs. These facial patterns were determined by the angle formed using the following cephalometric measurements: 1) Mandibular plane - the angle between the anterior cranial base (sella to nasion) and Mandibular plane (gonion to menton) - patients between 29 to 36 degrees were classified as normodivergent; patients with more than 36 degrees, as hyperdivergent; and less than 29 degrees, as hypodivergent;^12^ 2) Face height index - the ratio of posterior face height to anterior face height, using the measurements of distance from sella (S) to gonion (Go) divided by the distance of nasion (N) to menton (Me) - ratios of < 61%, 61% to 69%, and > 69% indicated hyperdivergent, normodivergent and hypodivergent patterns, respectively^13^ (Fig 1 and Table 1). 

**Table 1:** Average (X) and standard deviation (SD) of the distribution, by vertical facial patterns.

	VERTICAL FACIAL PATTERN			
DIMENSIONS	Normodivergent	Hyperdivergent	Hypodivergent	
	X (SD)	X (SD)	X (SD)	Total
Patients (n)	25	25	25	75
SN/GoMe (degrees)	33.26 (3.12)	42.42 (2.94)	20.71 (3.58)	
PFH/AFH (%)	66.90 (2.13)	55.87 (2.79)	82.35 (4.56)	

Subjects had to fit into a single facial pattern category for both measurements, in order to be included in the study. 

The normodivergent pattern group consisted of 16 men and 9 woman with an average age of 25.88 years; the hyperdivergent pattern group, by 14 men and 11 woman, with an average age of 24.04 years; and hypodivergent pattern group, by 15 men and 10 women, with an average age of 25.84 years.

The study protocol was approved by the Ethnical Committee of Peruvian Cayetano Heredia University (493-23-15).

All CBCTs were obtained from the Picasso Master CBCT (Vatech, Hwaseong, Korea), from the archives of patients previously treated for diagnostic reasons at the Section of Orthodontics, during the period of 2010 to 2016. The following settings were used: 120 kVp, 5 mA, scan time of 24 seconds, large field of view (20 cm x 19 cm), with a voxel size of 0.3mm. The three-dimensional (3D) images werere constructed using the Real Scan, version 2.0 software (Seoul, Korea). An orthodontist trained in using the software analyzed all CBCTs. 

All images were oriented in the standardized position before performing the measurements. In the axial view, the coordinate axis was placed at the midpoint between the infraorbital hole and the external ear canal, increasing the thickness of the image to 30mm so that both structures could be seen in the sagittal view. In the sagittal view, the tomographic volume was positioned in such a way that the Frankfort plane (Porion-Orbital) was parallel to the lower edge of the window. After locating the incisive foramen and posterior nasal spine (PNS) in the axial view, a reference line was constructed across the midpalatal suture. In the sagittal view, a midsagittal reference line was then projected through the distal margin of the incisive foramen and PNS.

**Figure 2:**
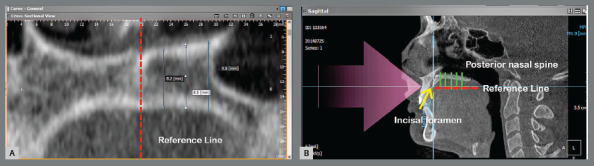
A) Coronal view: a reference line was constructed across the midpalatal suture. B) Sagittal view: a midsagittal reference line was then projected through the distal margin of the incisive foramen and PNS.

All subsequent measurements were made perpendicular to this reference line^9-15^ (Fig 2).

**Figure 3:**
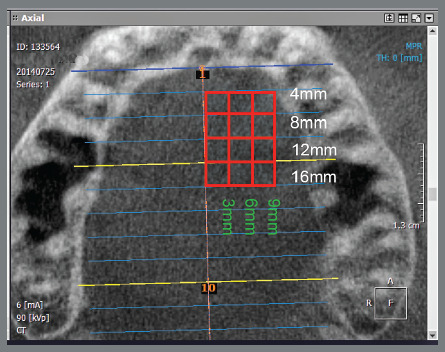
Measurement points at 3, 6 and 9mm lateral to midpalatal suture and 4, 8, 12 and 16mm posterior to the incisive foramen.

Measurements were taken at 4, 8, 12, 16 and 20mm posterior to the incisive foramen and were designated as P4, P8, P12, P16 and P20, respectively. Measurements taken at 3, 6 and 9mm lateral to midpalatal suture were designated as D3, D6 and D9, respectively. A total of 15 measurements were performed for each patient (Fig 3). Several studies used these measurements to evaluate the palatal bone before the installation of TADs.^9^ ^,^ ^11^ ^,^ ^12^

To evaluate the reliability of the method, the same examiner measured ten randomly selected subjects for all points, with a two week interval between trials. Intraclass correlation coefficient (ICC) was 0.92, showing an acceptable intraobserver agreement of repeated measurements. The inter-examiner reliability was evaluated between a dental radiologist and the principal examiner. Results showed a high correlation of 0.89. The results were evaluated at the significance level *p*< 0.05, with a 95% confidence interval.

The measurement error for the height and thickness of cortical palatal bone was 0.077 and 0.063mm, respectively. The measurement errors for the minimum and maximum values of cortical density of palatal bone were 35.92 and 21.69 attenuation coefficients, respectively.

The attenuation coefficient is a numerical value expressing the degree of attenuation producing body tissues on the x-ray beam. Higher values indicate high density anatomical tissues and lower values indicate low density tissue. The CBCT uses the attenuation coefficient to express the density in a grayscale. These measurements were made through an option to express the density in each CBCT, which are not standardized in the different equipment.

Statistical analysis was performed using SPSS 23 version software for Windows (IBM, Armonk, NY). Preliminary data analysis showed normal frequency distribution of the sample (Shapiro-Wick test). Descriptive statistics, ANOVA test with Tukey *post-hoc* test, was used for analysis of the data at a significance level of α = 0.05), with a 95% confidence interval, considering a test power of 80%.

## RESULTS

### PALATAL BONE HEIGHT

Comparison of palatal bone height measurements among the three vertical facial dimensions revealed that the hypodivergent group had the largest height values in D3/P4, D3/P8, D3/P12, D6/P12, D3/P16, D3/P20 and D6/P20, followed by the hyperdivergent and normodivergent groups (Table 2) (*p*< 0.05).

**Table 2:** Average (X) and standard deviation (SD) of the palatal bone height, by vertical facial patterns.

DIMENSIONS	VERTICAL FACIAL PATTERN						
	Normodivergent	Hyperdivergent	Hypodivergent	P*	P**		
	X (SD) mm	X (SD) mm	X (SD) mm		Normo/Hyper	Normo/Hypo	Hyper/Hypo
D3/P4	7.86 (2.28)	9.23 (3.16)	9.77 (2.78)	.048	.086	.011	.525
D6/P4	8.44 (2.72)	10.12 (3.38)	10.12 (3.00)	.084	.057	.043	.996
D9/P4	10.09 (2.92)	12.21 (3.94)	11.81 (2.71)	.054	.035	.036	.675
D3/P8	5.78 (2.01)	7.01 (3.20)	8.08 (2.74)	.013	.111	.001	.208
D6/P8	6.04 (2.41)	7.26 (3.43)	8.03 (2.97)	.064	.154	.012	.400
D9/P8	7.69 (2.70)	8.92 (3.65)	9.08 (2.97)	.234	.180	.089	.869
D3/P12	4.45 (1.53)	5.48 (2.66)	6.74 (2.45)	.003	.100	.000	.086
D6/P12	4.38 (1.96)	5.24 (2.60)	6.18 (2.58)	.035	.191	.008	.210
D9/P12	5.71 (2.42)	6.81 (3.21)	7.06 (2.42)	.178	.176	.054	.759
D3/P16	3.76 (1.46)	4.30 (1.91)	5.84 (2.49)	.001	.263	.001	.018
D6/P16	3.61 (1.74)	4.24 (2.20)	4.90 (2.16)	.089	.269	.024	.287
D9/P16	4.62 (2.07)	5.33 (2.63)	5.64 (2.49)	.307	.290	.119	.668
D3/P20	3.36 (1.29)	3.54 (1.46)	5.24 (2.32)	.000	.639	.001	.003
D6/P20	3.13 (1.36)	3.15 (1.57)	4.18 (2.01)	.046	.954	.036	.050
D9/P20	3.86 (1.60)	4.25 (2.17)	4.86 (2.43)	.245	.475	.093	.356

*: ANOVA test. **: *t*-Student test. D: Lateral to midpalatal suture / P: Posterior to the incisive foramen. mm: millimeters. 3, 4, 6, 8, 9, 12, 16, 20: Distance in millimeters.

The hyperdivergent group had significantly thicker palatal height (12.21 ± 3.94mm), compared to the normodivergent group (10.09 ± 2.92mm) in one place (D9/P4) (*p*< 0.05). When comparing hyperdivergent and hypodivergent groups, statistically significant differences were found in two places (D3/P16 and D3/P20) (*p*< 0.05), with a greater height in the hypodivergent group (5.84 ± 2.49mm *vs* 5.24 ± 2.32mm).

Comparing normodivergent and hypodivergent groups, the hypodivergent group obtained the largest dimensions, with statistically significant differences in D3/P4, D6/P4, D9/P4, D3/P8, D6/P8, D3/P12, D6/P12, D3/P16, D6/P16, D3/P20 and D6/P20, with an average difference of 2mm between the normodivergent and hypodivergent groups (*p*< 0.05).

### CORTICAL THICKNESS (PALATAL BONE)

Statistically significant differences were observed in most locations, these being D3/P4, D6/P4, D9/P4, D3/P8, D6/P8, D3/P12, D6/P12, D3/P16, D6/P16, D9/P16 and D3/P20 (*p*< 0.05). The hypodivergent group had the largest cortical thickness of palatal bone, followed by the hyperdivergent group and finally the normodivergent group, except for D9/P16, where the hyperdivergent group was greater than the hypodivergent group (Table 3).

**Table 3:** Average (X) and standard deviation (SD) of the cortical thickness of palatal bone, by vertical facial patterns.

DIMENSIONS	VERTICAL FACIAL PATTERN						
	Normodivergent	Hyperdivergent	Hypodivergent	P*	P**		
	X (SD) mm	X (SD) mm	X (SD) mm		Normo/Hyper	Normo/Hypo	Hyper/Hypo
D3/P4	2.07 (0.54)	2.44 (0.76)	2.90 (0.87)	.001	.054	.000	.050
D6/P4	2.11 (0.66)	2.37 (0.79)	2.86 (0.79)	.003	.219	.001	.031
D9/P4	2.15 (0.64)	2.55 (1.03)	2.99 (0.75)	.003	.109	.000	.088
D3/P8	1.69 (0.45)	2.01 (0.67)	2.37 (0.70)	.001	.057	.000	.070
D6/P8	1.78 (0.55)	1.82 (0.71)	2.28 (0.71)	.015	.806	.007	.027
D9/P8	1.92 (0.56)	2.12 (0.90)	2.30 (0.78)	.216	.334	.054	.474
D3/P12	1.42 (0.33)	1.69 (0.50)	1.96 (0.39)	.000	.032	.000	.035
D6/P12	1.35 (0.40)	1.56 (0.60)	1.90 (0.53)	.001	.155	.000	.036
D9/P12	1.57 (0.46)	1.78 (0.59)	1.87 (0.46)	.104	.161	.024	.558
D3/P16	1.26 (0.51)	1.62 (0.55)	1.76 (0.39)	.002	.023	.000	.291
D6/P16	1.14 (0.45)	1.44 (0.47)	1.45 (0.37)	.021	.026	.011	.947
D9/P16	1.35 (0.48)	1.64 (0.46)	1.62 (0.38)	.040	.032	.035	.814
D3/P20	1.28 (0.51)	1.39 (0.44)	1.75 (0.42)	.002	.425	.001	.005
D6/P20	1.16 (0.45)	1.26 (0.40)	1.42 (0.27)	.056	.395	.017	.106
D9/P20	1.26 (0.46)	1.48 (0.41)	1.53 (0.43)	.071	.086	.036	.640

*: ANOVA test. **: *t*-Student test. mm: millimeters. D: Lateral to midpalatal suture / P: Posterior to the incisive foramen. 3, 4, 6, 8, 9, 12, 16, 20: Distance in millimeters.

Comparing the average values of cortical thickness of the palatal bone between the normodivergent group and the hyperdivergent group, statistically significant differences were found in D3/P12, D3/P16, D6/P16, and D9/P16 (*p*< 0.05). The hyperdivergent group had a thicker cortical plate, compared to the normodivergent group (Table 3).

The hypodivergent group had a thicker cortical plate, compared to the hyperdivergent group, with a statistically significant difference (*p*< 0.05) in D6/P4, D6/P8, D3/P12, D6/P12 and D3/P20 (Hypo, 1.75 ± 0.42mm / Hyper, 1.39 ± 0.44mm). 

Comparing the average values of cortical thickness of the palatal bone between the normodivergent and hypodivergent groups, statistically significant differences (*p*< 0.05) were found in almost all places (D3/P4, D6/P4, D9/P4, D3/P8, D6/P8, D3/P12, D6/P12, D9/P12, D3/P16, D6/P16, D9/P16, D3/P20, D6/P20 and D9/P20). It was observed that the hypodivergent group has a thicker cortical (0.5mm to 1mm, in average) than the normodivergent group (Table 3). 

### CORTICAL DENSITY (PALATAL BONE)

No statistically significant differences were found in any of the locations indicated in the data collection sheet (Table 4 and Table 5).

**Table 4:** Average (X) and standard deviation (SD) minimum values of the cortical density palatal bone, by vertical facial patterns.

DIMENSIONS	VERTICAL FACIAL PATTERN						
	Normodivergent	Hyperdivergent	Hypodivergent	P*	P**		
	X (SD) AC	X (SD) AC	X (SD) AC		Normo/Hyper	Normo/Hypo	Hyper/Hypo
D3/P4	1262.56 (383.81)	1151.2 (440.16)	1227.84 (393.52)	.614	.345	.754	.519
D6/P4	1222.68 (392.46)	1077.76 (362.23)	1218 (393.60)	.321	.181	.967	.196
D9/P4	1246.76 (413.74)	1186.4 (417.81)	1209.92 (372.74)	.867	.610	.742	.835
D3/P8	1213.64 (391.57)	1079.12 (369.77)	1258.36 (429.70)	.259	.218	.702	.121
D6/P8	1137.68 (330.43)	1056.56 (427.11)	1193.24 (363.02)	.437	.456	.574	.229
D9/P8	1168.44 (361.66)	1141.04 (465.76)	1179.88 (416.96)	.944	.817	.918	.757
D3/P12	1199.72 (442.76)	1129.16 (463.76)	1262.32 (406.59)	.564	.585	.605	.286
D6/P12	1129.16 (427.25)	1076.48 (446.42)	1186.84 (381.61)	.650	.672	.617	.352
D9/P12	1088.84 (437.99)	1102.56 (419.26)	1133.48 (409.22)	.929	.910	.711	.793
D3/P16	1129.12 (414.20)	1134.28 (398.52)	1241.96 (395.49)	.538	.964	.329	.342
D6/P16	1127.68 (387.05)	995.4 (450.33)	1194.52 (480.46)	.274	.271	.591	.137
D9/P16	1124.6 (403.78)	1066.72 (421.44)	1161.84 (453.01)	.730	.622	.760	.446
D3/P20	1078.24 (432.33)	1133.64 (478.03)	1258.76 (403.80)	.336	.669	.134	.332
D6/P20	1075.44 (373.08)	1074.32 (468.68)	1146.56 (373.53)	.773	.993	.504	.550
D9/P20	1057.28 (398.45)	1027.04 (479.99)	1107.36 (422.44)	.805	.810	.668	.533

*: ANOVA test. **: *t*-Student test. D: Lateral to midpalatal suture / P: Posterior to the incisive foramen.

3, 4, 6, 8, 9, 12, 16, 20: Distance in millimeters. AC: Attenuation coefficient.

**Table 5:** Average (X) and standard deviation (SD) maximum values of the cortical density palatal bone by vertical facial patterns.

DIMENSIONS	VERTICAL FACIAL PATTERN						
	Normodivergent	Hyperdivergent	Hypodivergent	P*	P**		
	X (SD) AC	X (SD) AC	X (SD) AC		Normo vs Hyper	Normo vs Hypo	Hyper vs Hypo
D3/P4	1365.68 (420.71)	1254.92 (499.94)	1341.44 (403.44)	.651	.401	.836	.504
D6/P4	1317.8 (415.63)	1179.28 (403.32)	1317.36 (400.64)	.386	.238	.997	.231
D9/P4	1333 (447.63)	1293.76 (436.77)	1271 (382.76)	.872	.238	.601	.845
D3/P8	1304.08 (386.19)	1180.76 (389.41)	1353.68 (439.03)	.306	.755	.673	.147
D6/P8	1212.32 (354.49)	1139.8 (422.48)	1288.56 (396.00)	.411	.755	.477	.205
D9/P8	1287.8 (399.10)	1252.48 (491.68)	1285.28 (420.52)	.951	.266	.983	.801
D3/P12	1308.88 (441.72)	1235.28 (468.37)	1359.96 (414.27)	.607	.266	.675	.324
D6/P12	1241.36 (441.20)	1179.88 (437.19)	1284.8 (407.69)	.687	.514	.719	.385
D9/P12	1201.44 (435.75)	1206.2 (462.81)	1232.52 (429.56)	.965	.514	.801	.836
D3/P16	1224.96 (402.44)	1242.2 (434.71)	1348.6 (431.87)	.538	.782	.300	.390
D6/P16	1223.76 (416.59)	1141.8 (458.92)	1323.12 (456.68)	.358	.782	.426	.168
D9/P16	1228.32 (416.45)	1186.96 (437.29)	1242.44 (477.07)	.900	.570	.912	.670
D3/P20	1188 (422.72)	1293.08 (501.00)	1303 (413.44)	.604	.570	.336	.939
D6/P20	1161.6 (390.77)	1218.72 (468.34)	1262.2 (420.59)	.707	.623	.385	.731
D9/P20	1197 (411.34)	1244.44 (468.09)	1179.96 (425.76)	.864	.623	.886	.613

*: ANOVA test. **: *t*-Student test. D: Lateral to midpalatal suture / P: Posterior to the incisive foramen.

3, 4, 6, 8, 9, 12, 16, 20: Distance in millimeters. AC: Attenuation coefficient.

## DISCUSSION

The purpose of this study was to use CBCT to evaluate whether there is a difference in height, cortical thickness and density of the palatal bone in the different vertical facial patterns.

To evaluate the palatal bone and facial patterns, 3D images offer greater accuracy, compared with two-dimensional images, with high magnification and distorstion.^8^ ^,^ ^10^ ^,^ ^12^ Also, cephalograms reconstructed from the CBCT have no statistically significant differences on linear and angular measurements in relation to the traditional cephalograms and cranial physical measurements.^14^ Due to the existence of diverse studies that have demonstrated the accuracy of the CBCT, the present study used these 3D volumes for the evaluation of facial patterns and the palatal bone.^15^ ^,^ ^16^

In the present study, the hypodivergent pattern presented a higher height and greater thickness of the cortical palatal bone, compared to the normodivergent and hyperdivergent patterns. However, no statistically significant differences were found in the values of cortical density. The findings of this study could be attributed to the adaptation of the palatal bone, influenced by numerous genetic and environmental factors, which are detailed below.

### PALATAL BONE HEIGHT

Several studies reported that there are statistically significant differences when comparing the height of the dentoalveolar process in the maxilla and mandible in patients with different facial patterns.^10,16-19^ Sadek et al.,^10^ using CBCT, reported that hyperdivergent patients had a greater dentoalveolar height in the anterior section, both in the upper and lower jaw, followed by normodivergent and finally the hypodivergent patterns.

In the present study, by measuring the height of the palatal bone, statistically significant differences were found between the facial patterns. However, the hypodivergent sample had a greater palatal bone height, followed by hyperdivergent and normodivergent patterns. Sadek et al.^10^ found different results, where the dentoalveolar process in the upper and lower jaw is influenced not only by genetic factors, but also by the dentoalveolar adaptation process against different loads of oral and perioral muscle strength.^10^ ^,^ ^16^ ^-^ ^19^

For example, the tongue activity pattern during the swallowing and breathing can affect the morphological development of the palatal bone.^19^

During the process of growth and development, the palatal bone in normal situations suffers a process of remodelling, with respect to its height, due to the resorption in the nasal chambers and bone-apposition on the buccal side of the palate, suggesting that different breathing patterns (nasal or naso-buccal) could alter the height of the palatal bone.^19^ These differences could affect the palatal bone dimensions, according to the Kang et al.^20^ study (CBCT scans of children, 27 mouth breathers and 27 nose-breathers), who concluded that mouth breathers may have less palatal support tissues than nose breathers, because the majority of mouth breathers have a high-angle pattern in the vertical direction.

These physiological events could explain the present results, by comparing the average values of the height of the palatal bone between pairs (normo/hyper, normo/hypo and hyper/hypo), where the hypodivergent pattern had a greater palatal bone height. Similarly, Flores-Blancas et al.^21^ (99 lateral cephalograms of post-pubertal individuals), found that brachifacial patterns had greater nasopharyngeal widths, compared to other vertical facial patterns, and that these changes could be influenced by the craniofacial growth pattern.

In addition, Hwang et al. ^22^ (CBCT scans of 101 adults aged 22 to 26 years) related the masticatory muscles and craniofacial growth. Likewise, the muscular hyperactivity of the hypodivergent patterns produces an increase in the mechanical load that would generate a greater bone apposition. On the contrary, hyperdivergent patterns show narrow and deep palates due to a weak muscular pattern.^22^

### CORTICAL THICKNESS (PALATAL BONE)

Several CBCT studies reported no statistically significant differences on cortical thickness and density when these were measured on both sides of the palatal bone.^1,23-27^ Baumgaertel et al.^25^ (CBCT scans of 30 adults dry skulls) and Kang et al.^26^ (CT records of 18 adults aged 18 to 35 years) found no significant differences between the thickness of the cortical bone on the right and left sides.

Ozdemir et al.^24^ (CBCTs of 155 patients, aged 20 to 45 years) evaluated the cortical thickness of the alveolar process from the buccal side of the jaw and the palatal alveolar process in the maxilla in patients with different vertical patterns. They observed a greater cortical thickness in hypodivergent patients, compared to normodivergent and hyperdivergent patients.

There are few studies linking cortical thickness with the vertical patterns. Matsumoto et al.^27^ (CTs of 31 dry skulls, aged 18 to 45 years) and Tsunori et al.^28^ (CTs of 39 dry skulls of male Asiatic Indians) found no correlation between facial type and cortical thickness of the jaws.

Johari et al.^14^ (CBCT of patients in permanent dentition) evaluated the relationship between the thickness of the cortical area of the mid-palatal suture and facial height. They concluded that hypodivergents had greater cortical thickness than normodivergents and hyperdivergents. They also found no statistically significant differences when comparing the normo and hyperdivergent groups, similar to the present results, which show that the hypodivergent pattern also had a greater thickness of the palatal cortical bone. However, in the Johari et al.^14^ study, proportionality on the number of patients was not kept, unlike the present study, which had an equal number for each facial pattern.

### CORTICAL DENSITY (PALATAL BONE)

No statistically significant differences were found in any of the vertical facial patterns.

Han et al.^9^ reported a higher density of cortical and trabecular bones in adults, compared to teenagers, in CBCTs. These measurements were presented in Hounsfield units (HU), which differed from the present study, which used attenuation coefficients.

Moon et al.^11^ and Han et al.^9^ found a higher density in women. Furthermore, the palatal bone density tends to decrease from the anterior to the posterior area and from the midpalatal suture to the paramedian areas. No significant differences in cortical density of the palatal bone between the anterior and posterior sectors were found in the present study. Thus when comparing with Moon et al.,^11^ it can be mentioned that data obtained from CT scanners cannot be extrapolated to CBCTs.Similarly, Ozdemir et al.^8^ found no significant differences in the cortical density of the palatine bone between the right and left sides between the dentoalveolar buccal and palatal areas.

According to different published studies, most of these use CBCT and express the cortical density in HU, using the correct term: attenuation coefficient - the unit indicated to express the cortical density.^29^ ^,^ ^30^

**Figure 4:**
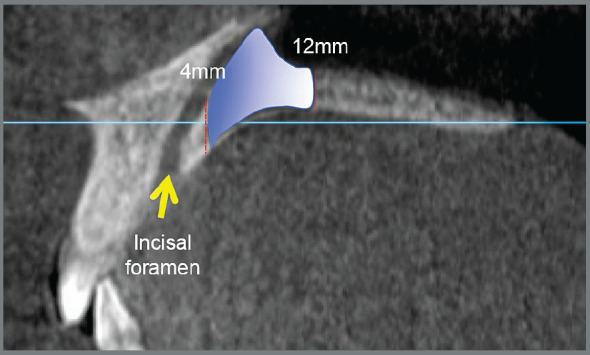
Hypodivergent patterns. Area between 4 and 12 mm posterior to the incisal foramen. Maximum height and cortical thickness of 11.81mm and 2.99mm, respectively (canine distal approx.), minimum cortical height and thickness of 6.18mm and 1.87mm, respectively (second premolar distal approx.).

Based on the results of the present study, the following clinical recommendations can be made: In patients with hypodivergent pattern, it is suggested to install TADs in the area between 4 and 12mm posterior to the incisal foramen and 3 to 9mm lateral to the middle palatal suture. This area has dimensions of maximum height and cortical thickness of 11.81mm/2.99mm respectively (canine distal approx.) and minimum cortical height and thickness of the palatal bone of 6.18mm, 1.87mm respectively (second premolar distal approx.), as seen in Figure 4.

**Figure 5:**
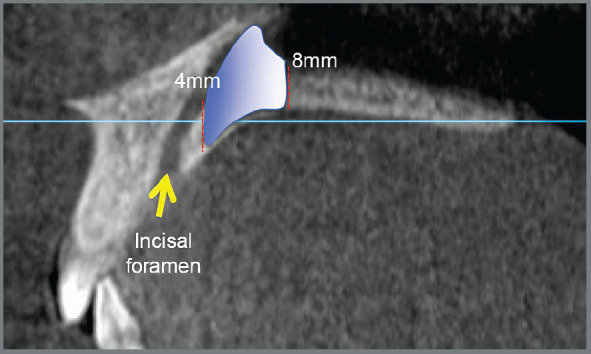
Normodivergent and hyperdivergent patterns. Area between 4 and 8 mm posterior to the incisal foramen. Maximum height and cortical thickness of 12.21mm and 1.69mm, respectively (canine distal approx.); minimum cortical height and thickness of 5.78mm and 2.55mm, respectively (first premolar distal approx.).

No statistically significant differences were found in patients with normodivergent and hyperdivergent patterns, being suggested the installation of TADs in the area between 4 and 8 mm posterior to the incisal foramen and 3 to 9mm lateral to the midpalatal suture. This area has dimensions of maximum height and cortical thickness of 12.21mm and 1.69mm, respectively (canine distal approx.); and minimum cortical height and thickness of the palatal bone of 5.78mm and 2.55mm, respectively (first premolar distal approx.), as seen in Figure 5.

Investigations comparing the dimensions of the palatal bone and vertical facial patterns, gender and age group, are suggested as a complement to this investigation.

## CONCLUSION

The palatal bone is a favorable anatomical area to install different orthodontic temporary anchorage devices (TADs) where individuals with the hypodivergent vertical facial pattern have a higher height and cortical thickness of the palatal bone, followed by the hyperdivergent pattern and finally the normodivergent pattern. 

Likewise, no statistically significant differences for the cortical density of the palatal bone were found between the three vertical facial patterns.
